# Predictors of Intelligence at the Age of 5: Family, Pregnancy and Birth Characteristics, Postnatal Influences, and Postnatal Growth

**DOI:** 10.1371/journal.pone.0079200

**Published:** 2013-11-13

**Authors:** Hanne-Lise Falgreen Eriksen, Ulrik Schiøler Kesmodel, Mette Underbjerg, Tina Røndrup Kilburn, Jacquelyn Bertrand, Erik Lykke Mortensen

**Affiliations:** 1 Department of Public Health, Section of Epidemiology, Aarhus University, Aarhus, Denmark; 2 Department of Obstetrics and Gynaecology, Aarhus University Hospital, Aarhus, Denmark; 3 Children’s Neurocenter at Vejlefjord Rehabilitation Center, Stouby, Denmark; 4 Centers for Disease Control and Prevention (CDC), Atlanta, Georgia, United States of America; 5 Department of Public Health and Center for Healthy Ageing, University of Copenhagen, Copenhagen, Denmark; Hôpital Robert Debré, France

## Abstract

Parental education and maternal intelligence are well-known predictors of child IQ. However, the literature regarding other factors that may contribute to individual differences in IQ is inconclusive. The aim of this study was to examine the contribution of a number of variables whose predictive status remain unclarified, in a sample of basically healthy children with a low rate of pre- and postnatal complications. 1,782 5-year-old children sampled from the Danish National Birth Cohort (2003–2007) were assessed with a short form of the Wechsler Preschool and Primary Scale of Intelligence – Revised. Information on parental characteristics, pregnancy and birth factors, postnatal influences, and postnatal growth was collected during pregnancy and at follow-up. A model including study design variables and child’s sex explained 7% of the variance in IQ, while parental education and maternal IQ increased the explained variance to 24%. Other predictors were parity, maternal BMI, birth weight, breastfeeding, and the child’s head circumference and height at follow-up. These variables, however, only increased the explained variance to 29%. The results suggest that parental education and maternal IQ are major predictors of IQ and should be included routinely in studies of cognitive development. Obstetrical and postnatal factors also predict IQ, but their contribution may be of comparatively limited magnitude.

## Introduction

Psychometric intelligence (IQ) as a measure of general cognitive ability is a major predictor of important outcomes across the lifespan, such as socioeconomic status and health [1], [2]. It is therefore relevant to identify the factors that determine individual differences in IQ. A large body of empirical evidence across various types of study populations consistently points to parental social position, parental education, maternal intelligence, and (early) home environment as significant predictors of IQ [3], [4]. Parental education has been shown to account for 19% of the variance in child IQ [5], while maternal IQ and home environment in combination have been shown to account for 25–29% [6].

Additional factors whose predictive power has been studied include biomedical risk factors (e.g., low birth weight [7] and prematurity [6]), prenatal/early exposures impacting fetal or postnatal development of the central nervous system (e.g., maternal drug use in pregnancy or environmental pollutants [8]), and nutritional factors (e.g., breastfeeding, specific food components, and general nutrition [9], [10]). However, both positive and negative findings have been reported in such studies, and in several cases the effects of a particular risk factor are non-significant or substantially reduced after adjusting for parental education, maternal IQ and/or home environment [9]. The different findings may reflect the fact that the variance explained by a risk factor in a given study populations depends not only on its strength, but also on the prevalence and on the interaction of the risk factor with other factors influencing development.

The primary objective of the present study was to conduct a systematic evaluation of a broad selection of both well-established and less well-investigated predictors of IQ in a large sample of basically healthy, 5-year-old children selected from the Danish National Birth Cohort (DNBC). Availability of a variety of potential predictor variables made it possible to estimate the relative contribution of each individual predictor, while taking into account other known and potential explanatory factors. In particular, the aim was to identify variables that explained variance in addition to the variance explained by maternal IQ and parental education in this non-clinical sample, with potential implications for the design and choice of covariates in studies of developmental influences on intelligence.

## Methods

### Ethics Statement

The study received permission from The Central Denmark Region Committee on Biomedical Research Ethics and all mothers provided written informed consent.

### Participants

The study sample comprised 1,782 mother-child pairs participating in the Lifestyle During Pregnancy Study (LDPS) [11], a comprehensive follow-up study of prenatal, lifestyle-related exposures (primarily maternal alcohol consumption) and children’s cognitive and motor abilities at the age of 5 years. Participants in the LDPS were sampled from the Danish National Birth Cohort (DNBC), which comprises information on the pregnancies of 101,042 Danish women [12]. Sampling procedure was based primarily on maternal intake of alcohol during pregnancy as reported in a prenatal telephone interview. In the interview, the women were asked about their average weekly intake of alcohol and number of binge drinking episodes (i.e. 5 or more units on a single occasion) and sampled into different categories of alcohol consumption (primarily 0, 1–4, 5–8 and 9 or more units per week). The higher exposure categories were oversampled and all statistical analyses were weighted by sampling probabilities. For a full description of the sampling design see [13]. The average weekly intake in the LDPS sample was low (median = ½ drink) and did not exceed 7 units for the majority of the women (97.9%).

Exclusion criteria were: multiple pregnancies; inability to speak Danish; impaired hearing or vision likely to compromise the ability to perform the cognitive tests, and congenital disabilities that imply or are likely to imply mental retardation (e.g. trisomy 21 or infantile autism).

### Procedure

Between September 2003 and June 2008, 3,478 women were invited to participate in the LDPS when their children were 60–64 months of age. Of those invited, 1,782 (51.0%) participated in a comprehensive follow-up assessment, which included administration of IQ tests to both the mother and the child. The assessments were carried out at test sites located in Copenhagen, Odense, Aalborg and Aarhus, hence covering all regions of Denmark. Test procedures were standardized in detail and carried out by 10 trained psychologists.

### Outcome Variable

IQ was assessed with the Wechsler Primary and Preschool Scales of Intelligence - Revised (WPPSI-R) [14]. The WPPSI-R comprises five verbal subtests and five performance subtests from which Verbal IQ (VIQ), Performance IQ (PIQ), and Full-Scale (FSIQ) IQs are derived. The short form used in the present study included three verbal subtests (Arithmetic, Information, and Vocabulary) and three performance subtests (Block Design, Geometric Design, and Object Assembly). All 6 subtests were completed by 1,769 children, whereas 13 children completed at least two verbal and two performance subtests, which was the minimum for prorating IQs using standard procedures. Since no Danish WPPSI-R norms were available at the time of the study, Swedish norms were used to derive scaled scores and IQs [15], and consequently the observed distribution of IQ in the sample does not necessarily correspond to the expected theoretical mean of 100 and standard deviation (SD) of 15.

### Predictor Variables

#### Study design variables (codings in the statistical analyses shown in parentheses)

Potential tester effects for the WPPSI-R were taken into account by the inclusion of an indicator variable for testing psychologist. Well-normed IQ scores are age-adjusted, but because Swedish norms were used, the age of the child at the time of testing was categorized in four 1-month age-bands (i.e. 60 to <61 months, 61 to <62 months, etc.) and included in all models.

#### Parental characteristics

Detailed information on parental education was obtained by a questionnaire completed by the parents at follow-up. Educational level was derived as the sum of years in school plus years of post-school theoretical education. The average score of both parents was used, if available, otherwise the mother’s score was used (39 cases).

Maternal IQ was assessed at the follow-up with two verbal subtests (Information and Vocabulary) from the Wechsler Adult Intelligence Scale (WAIS) [16] and with the non-verbal Raven’s Standard Progressive Matrices [17]. The raw scores of each test were standardized based on the results from the full sample and weighted equally in a combined score that was restandardized to a full IQ scale with a mean of 100 and an SD of 15.

Information on maternal age in years was obtained from the Danish civil registration system. Paternal age in years, parity (0, 1, or 2+ previous pregnancies), maternal pre-pregnancy Body Mass Index (BMI; weight in kg/(height in m)^2^), and maternal prenatal marital status (single, married/cohabiting) was obtained from the DNBC.

#### Pregnancy and birth characteristics

The child’s sex and date of birth were obtained from the Danish civil registration system. Information on gestational age in days (based on the last menstrual period), birth weight (grams), birth length, head circumference (cm), and Apgar score at 5 minutes (<7, ≥7) was obtained from the Danish Birth Registry. Information on maternal alcohol consumption and smoking during pregnancy was obtained from the DNBC at a median of 17 weeks of gestation and included as binary variables in the statistical analyses.

#### Postnatal influences

Information on maternal postnatal marital status (single, married/cohabiting) was obtained from the follow-up questionnaire, as was information on breastfeeding (≤1 month, >1 month) and postnatal parental smoking (none/either or both parents smokers). No validated Danish home environment index was available, so the following information from the follow-up questionnaire was included as indicators of a suboptimal home environment: daycare for 8+ hours/day before age three, 14+ days’ separation from parents (e.g. due to hospital admission or foster home), irregular breakfast (child is not always served breakfast), maternal depression (saw a doctor about depressive symptoms), and high maternal or paternal alcohol consumption (>14 and >21 drinks/week for mothers and fathers respectively, corresponding to the recommended maximum intake from the Danish National Board of Health).

#### Postnatal growth

Postnatal growth parameters of the child (head circumference (cm), height (cm), and weight (kg)) were measured at the 5-year follow-up. BMI (weight in kg/(height in m)^2^) was calculated from this information.

### Analytic Approach

All statistical analyses were conducted with Stata 11 (StataCorp LP, College Station, Texas).

The number of missing values for individual variables ranged from 2 (maternal prenatal marital status) to 96 (breastfeeding), with 10 missing values on the IQ outcome variable. For most variables (19 of 26) the extent of missing values was below 1.2%. Missing values were imputed based on a dedicated model for imputations, for which variables were modelled from the other variables considered to be most predictive (specific equations are available upon request) and in which 100 completed data sets were generated. All conclusions were maintained when a complete case analysis was conducted (*n* = 1589–1747). We report results from the imputed analyses. All imputations were performed with the ice add-on command and the built-in mi estimate command of STATA 11 [18]. In the LDPS, higher alcohol categories were oversampled, and consequently, all analyses were weighted by sampling fractions.

First, Pearson correlations were used to evaluate bivariate associations between each potential predictor and FSIQ outcome (point-biserial correlation for binary predictors). Second, a series of linear regression analyses were conducted for each of the three IQ outcomes (FSIQ, VIQ, and PIQ). For each domain of potential predictors (parental characteristics, pregnancy and birth characteristics, postnatal influences, and postnatal growth), the predictive power of the included variables was evaluated in multiple linear regression models. In these analyses, all models included parental education, maternal IQ, the sex of the child, and the two study design variables (the child’s age and tester). These variables were considered core predictors and included in all models to obtain a reasonably realistic picture of the effects of other potential predictors in each domain.

Third, predictors with a *p*-value of 0.10 or below for any IQ-measure in the analyses of each predictor domain were included in a full regression model that also included the two core predictors, sex of the child, and the study design variables. This selection criterion was chosen to avoid excluding marginally significant factors that could potentially gain significance in a model with fewer covariates and less unexplained variance. All statistical tests were two-sided and declared significant at the 5% level.

Potential collinearity among the predictor variables was evaluated by calculating the multiple *R^2^* between each predictor variable and all other predictors. High multiple *R* ^2^s were obtained for birth weight and birth length (*R^2^* = 0.75 and 0.67); to avoid collinearity between these measures, only birth weight was included in the statistical models. *R^2^*s for the remaining predictor variables did not exceed 0.53 (maternal age).

Preliminary analyses showed that head circumference, height, and BMI at follow-up were quadratically related to FSIQ (adjusted for the core predictors). Consequently, quadratic terms were included for these variables in the model of postnatal growth, but only the quadratic term for head circumference was significant and therefore included in the final model.

## Results

### Sample Composition

Table 1 shows that the parents were relatively well-educated, relatively old and that the pregnancy was the first for about half of the mothers. A little more than one fourth of the mothers smoked during pregnancy while about half of the mothers consumed alcohol during pregnancy. Half of the children were males, and the mean gestational age and birth weight were inconspicuous.

**Table 1:** Study sample characteristics, Denmark 2003–2007.

Family background								
	Mean (SD)		*r*			*p*		
Parental education (years)	13.2 (1.9)		0.30			<0.001		
Maternal IQ	100.0 (15.0)		0.40			<0.001		
Maternal age (years)	30.8 (4.4)		0.05			0.183		
Paternal age (years)	33.3 (5.4)		0.02			0.478		
Parity	1.7 (0.8)		−0.08a,b			0.004		
*0 (%)*	50.9							
*1 (%)*	32.1							
*2+ (%)*	17.0							
Maternal BMI (kg/m^2^)	23.5 (4.0)		−0.12			0.002		
Single mother, prenatally (%)	3.2		−0.10			0.156		
**Pregnancy and birth**								
Sex *(Male %)*	51.9			−0.17^c^		<0.001		
Gestational age (days)	280.6 (10.6)			0.08		0.015		
Birth weight (grams)	3603.2 (515.2)			0.08		0.028		
Birth length (cm)	52.4 (2.3)			0.06		0.095		
Head circumference at birth (cm)	35.3 (1.6)			0.07		0.064		
Apgar score in 5 minutes <7 (%)	0.5			0.00		0.938		
Mother smoked in pregnancy (%)	28.4			−0.13		0.002		
Mother consumed alcohol in pregnancy (%)	52.3			0.03		0.435		
**Postnatal influences**								
Single mother (%)	11.1			−0.06		0.076		
Breastfeeding >1 month (%)	85.7			0.18		<0.001		
Parental smoking (%)	32.3			−0.07		0.039		
*Indicators of suboptimal home environment*								
Daycare 8+ hours/day before age 3 (%)		11.9			0.02		0.541	
14+ days’ separation from parents (%)		0.8			0.02		0.111	
Irregular breakfast meals (%)		4.3			−0.10		0.019	
Maternal depression (%)		19.1			−0.02		0.656	
Maternal alcohol consumption >14 drinks/week (%)		3.3			−0.05		0.275	
Paternal alcohol consumption >21 drinks/week (%)		4.9			−0.01		0.740	
**Postnatal growth**								
Head circumference (cm)		51.8 (1.5)			0.15^d^		<0.001	
Height (cm)		113.7 (4.4)			0.10^d^		0.001	
BMI (kg/m^2^)		15.8 (1.3)			−0.07b,d		0.007	

*N* = 1,772. For the dichotomous predictor variables, testing the significance of the point-biserial correlations is equivalent to testing the significance of mean differences on FSIQ between the two subsamples defined by the binary predictor.

a Multiple *R* for categories 1 and 2+.

b Technically, *R* is positive; here, the minus sign indicates the direction of the association.

c Reference category: Females.

d Multiple *R*’s for linear and quadratic variables.

### Bivariate Correlations

As shown in Table 1, the predictor variables that showed the strongest bivariate correlations with FSIQ test performance were maternal IQ, parental education, maternal BMI, maternal smoking in pregnancy, sex, breastfeeding, and head circumference at follow-up (*p* for all coefficients <0.01). Gestational age, birth weight, postnatal parental smoking, irregular breakfast meals, and height at five years were also significantly associated with FSIQ.

The Pearson correlation between Verbal IQ and Performance IQ was 0.46, while the correlations with Full-Scale IQ were 0.81 and 0.89, respectively (*p*<0.001 for all values). There were no substantial differences in the fit of the models between Verbal IQ and Performance IQ.

### Models of Predictor Domains

In the intermediate models analyzing domains of predictors, the significant predictors (*p*≤0.05) were birth weight, with a positive effect on FSIQ and PIQ; breastfeeding, which had a positive effect on all three IQ outcomes, while of the postnatal growth measures, head circumference was quadratically associated with FSIQ and PIQ, height was linearly associated with all three IQs and there were no significant effects of BMI. Maternal IQ, parental education and sex were significant predictors in all four predictor domains.

The following variables had a *p*-value ≤0.10 for at least one of the three IQ measures (see Table 2) and thus qualified for the final model: higher maternal age and single mother status were positively associated with VIQ; parity, maternal BMI, and paternal age were negatively associated with VIQ; irregular breakfast had a negative effect on FSIQ.

**Table 2:** Predictor variables and WPPSI-R scores within predictor domains, Denmark 2003–2007.

	Full scale IQ				Verbal IQ				Performance IQ		
Sample mean (SD)	105.5 (12.8)				104.8 (10.7)				105.0 (16.2)		
Parental characteristics^a^											
	*β*		95% CI	*P*	*β*		95% CI	*p*	*β*	95% CI	*p*
Parental education (years)	0.9		0.4; 1.3	<0.001	1.0		0.5; 1.4	<0.001	0.8	0.2; 1.4	0.012
Maternal intelligence (IQ points)	0.3		0.2; 0.3	<0.001	0.2		0.2; 0.3	<0.001	0.3	0.2; 0.4	<0.001
Child sex^b^	−3.7		−5.1; −2.1	<0.001	0.1		−1.3; 1.5	0.887	−7.4	−9.5; −5.3	<0.001
Maternal age (years)	0.0		−0.3; 0.3	0.998	0.2		0.0; 0.4	0.084	0.2	−0.6; 0.2	0.289
Paternal age (years)	−0.1		−0.3; 0.1	0.525	−0.2		−0.3; 0.0	0.051	0.0	−0.3; 0.3	0.796
Parity *0 vs 1*	−0.1		−1.8; 1.7	0.938	−1.2		−2.8; 0.4	0.151	1.0	−1.4; 3.5	0.407
*0 vs. 2+*	−0.6		−2.7; 1.5	0.551	−2.8		−4.8; −0.8	0.029	1.5	−1.5; 4.6	0.316
Maternal BMI (kg/m^2^)	−0.1		−0.3; 0.0	0.131	−0.2		−0.4; 0.0	0.007	−0.1	−0.3; 0.2	0.495
Single mother, prenatally	0.8		−2.5; 4.1	0.618	4.0		0.6; 7.4	0.022	−2.3	−8.6; 4.0	0.479
**Pregnancy and birth** a											
	***β***	**95% CI**		***p***	***β***		**95% CI**	***p***	***β***	**95% CI**	***p***
Parental education (years)	1.0	0.5; 1.4		<0.001	1.1		0.7; 1.6	<0.001	0.8	0.2; 1.4	0.014
Maternal intelligence (IQ points)	0.2	0.2; 0.3		<0.001	0.2		0.1; 0.2	<0.001	0.3	0.2; 0.4	<0.001
Child sex^b^	−3.9	−5.4; −2.5		<0.001	0.0		−1.4; 1.4	0.961	−7.8	−9.9; −5.7	<0.001
Gestational age (days)	0.0	−0.1; 0.1		0.583	0.1		0.0; 0.1	0.191	0.0	−0.1; 0.1	0.901
Birth weight (units of 100 grams)	0.2	0.0; 0.3		0.025	0.0		−0.1; 0.2	0.837	0.3	0.1; 0.5	0.002
Apgar score *<7*	1.2	−5.2; 7.5		0.715	1.9		−9.2; 13.1	0.733	0.4	−5.1; 5.9	0.882
Smoking in pregnancy	−0.6	−2.3; 1.1		0.494	−0.2		−1.9; 1.5	0.818	−0.9	−3.4; 1.4	0.414
Alcohol consumption in pregnancy	0.1	−1.4; 1.7		0.890	0.6		−0.9; 2.0	0.437	−0.4	−2.5; 1.8	0.752
**Postnatal influences** a											
	***β***	**95% CI**		***p***		***β***	**95% CI**	***p***	***β***	**95% CI**	***p***
Parental education	0.8	0.4; 1.3		<0.001		1.0	0.6; 1.5	<0.001	0.6	0.0; 1.2	0.056
Maternal IQ	0.2	0.2; 0.3		<0.001		0.2	0.1; 0.2	<0.001	0.3	0.2; 0.4	<0.001
Child sex	−4.0	−5.5; −2.5		<0.001		−0.2	−1.6; 1.2	0.768	−7.8	−9.9; −5.7	<0.001
Single mother	−0.7	−3.3; 1.8		0.576		−0.2	−2.5; 2.0	0.853	−1.2	−5.1; 2.6	0.526
Breastfeeding >1 month	3.9	1.2; 6.3		0.004		3.2	0.9; 5.4	0.005	4.5	0.8; 7.9	0.016
Parental smoking	−0.6	−2.3; 1.0		0.443		−0.5	−2.1; 1.0	0.482	−0.7	−3.0; 1.6	0.544
Daycare 8+ hours/day before age 3	−0.1	−2.4; 2.1		0.904		−1.0	−3.1; 1.2	0.385	0.6	−2.5; 3.9	0.677
14+ days’ separation from parents	1.7	−3.0; 6.5		0.475		0.5	−3.7; 4.7	0.812	2.9	−3.7; 9.6	0.386
Irregular breakfast meals	−3.8	−8.3; 0.7		0.099		−2.2	−5.8; 1.3	0.218	−5.3	−11.8; 1.1	0.104
Maternal depression	0.3	−1.4; 2.0		0.725		0.4	−1.3; 2.1	0.624	0.2	−2.3; 2.7	0.877
High maternal alcohol consumption	3.6	−4.7; 12.0		0.393		0.4	−6.2; 7.1	0.895	6.8	−4.8; 18.5	0.251
High paternal alcohol consumption	−0.7	−3.8; 2.5		0.682		1.3	−2.3; 4.8	0.487	−2.6	−7.2; 2.0	0.272
**Postnatal growth** a											
	***β***	**95% CI**		***p***		***β***	**95% CI**	***p***	***β***	**95% CI**	***p***
Parental education (years)	0.9	0.5; 1.3		<0.001		1.1	0.7; 1.6	<0.001	0.7	0.1; 1.3	0.025
Maternal intelligence (IQ points)	0.2	0.2; 0.3		<0.001		0.2	0.1; 0.3	<0.001	0.3	0.2; 0.4	<0.001
Child sex	−4.8	−6.3; −3.3		<0.001		−0.6	−2.0; 0.8	0.398	−9.0	−11.1; −6.9	<0.000
Head circumference (cm)	0.8	0.2; 1.3		0.010		0.3	−0.3; 0.8	0.354	1.3	0.4; 2.1	0.002
Head circumference squared	−0.3	−0.6; 0.0		0.045		−0.2	−0.4; 0.1	0.268	−0.4	−0.8; −0.1	0.018
Height (cm)	0.3	0.1; 0.4		0.008		0.3	0.1; 0.4	0.001	0.2	0.0; 0.5	0.082
Height squared	0.0	0.0; 0.0		0.173		0.0	0.0; 0.0	0.058	0.0	0.0; 0.0	0.508
BMI *(*kg/m^2^)	0.1	−0.6; 0.7		0.818		0.2	−0.5; 0.8	0.600	0.0	−1.0; 1.0	0.987
BMI squared	−0.2	−0.5; 0.0		0.095		−0.2	−0.4; 0.1	0.174	−0.2	−0.6; 0.1	0.137

WPPSI-R: Wechsler Preschool and Primary Scale of Intelligence-Revised.

a Regression coefficients and *p*-values adjusted for child age, sex, and tester.

b Reference category: females.

### Final Model

Table 3 presents the model of all predictors with a *p*-value ≤0.10 in the models of predictor domains. Parity and maternal BMI were negatively and prenatal marital status positively associated with VIQ (maternal BMI also with FSIQ), while birth weight was positively associated with FSIQ and PIQ. Breastfeeding was positively associated with all three IQ scales. Head circumference at 5 years was quadratically associated with FSIQ and PIQ, whereas height at 5 years was associated with outcomes on FSIQ and VIQ. Maternal and paternal age and irregular breakfast were not significant predictors in this model.

**Table 3:** Selected predictor variables and WPPSI-R scores, Denmark 2003–2007.

	Full scale IQ^a^					Verbal IQ^a^				Performance IQ^a^			
	*β*	95% CI		*p*	Part. *r* ^2^	*β*	95% CI	*p*	Part. *r* ^2^	*β*	95% CI	*p*	Part. *r* ^2^
Core predictors and sex													
Parental education (years)	0.7	0.3; 1.1	0.002		0.0056	0.8	0.4; 1.3	<0.001	0.0083	0.5	−0.1; 1.2	0.078	0.0018
Maternal IQ (IQ points)	0.3	0.2; 0.3	<0.001		0.0461	0.2	0.2; 0.3	<0.001	0.0329	0.3	0.2; 0.4	<0.001	0.0345
Child’s sex	−4.9	−6.3; −3.4	<0.001		0.0233	−0.6	−2.0; 0.8	0.396	0.0004	−9.1	−11.2; −7.0	<0.001	0.0385
Parental characteristics													
Maternal age (years)	0.0	−0.3; 0.2	0.743		0.0000	0.2	−0.1; 0.4	0.131	0.0013	−0.2	−0.6; 0.1	0.168	0.0011
Paternal age (years)	0.0	−0.2; 0.1	0.617		0.0001	−0.1	−0.3; 0.0	0.080	0.0018	0.0	−0.2; 0.3	0.729	0.0001
Parity *0 vs 1*	−0.6	−2.4; 1.1	0.485		0.0003	−1.5	−3.1; 0.1	0.072	0.0019	0.3	−2.2; 2.7	0.840	0.0000
*0 vs. 2+*	−1.4	−3.5; 0.7	0.185		0.0010	−3.3	−5.3; −1.2	0.002	0.0055	0.4	−2.6; 3.5	0.784	0.0000
Maternal BMI (kg/m^2^)	−0.2	−0.4; 0.0	0.029		0.0028	−0.2	−0.4; −0.1	0.009	0.0039	−0.2	−0.4; 0.1	0.177	0.0010
Prenatal single mother	0.3	−3.0; 3.6	0.856		0.0000	3.5	0.0; 6.9	0.050	0.0022	−2.8	−9.2; 3.5	0.381	0.0004
Pregnancy and birth													
Birth weight (units of 100 grams)	0.1	0.0; 0.3	0.048		0.0023	0.1	−0.1; 0.2	0.392	0.0004	0.2	0.0; 0.4	0.022	0.0030
Postnatal influences													
Breastfeeding	3.8	1.3; 6.4	0.003		0.0050	3.3	1.1; 5.5	0.004	0.0049	4.4	0.8; 8.0	0.017	0.0033
Irregular breakfast	−3.8	−8.1; 0.5	0.086		0.0017	−2.2	−5.7; 1.2	0.205	0.0009	−5.4	−11.5; 0.8	0.088	0.0017
Postnatal growth													
Head circumference (cm)	0.7	0.1; 1.2	0.017		0.0033	0.3	−0.2; 0.8	0.266	0.0007	1.1	0.3; 1.8	0.009	0.0040
Head circumference squared	−0.3	−0.6; 0.0	0.020		0.0031	−0.2	−0.4; 0.1	0.161	0.0011	−0.4	−0.8; −0.1	0.009	0.0039
Height (cm)	0.2	0.0; 0.4	0.031		0.0027	0.2	0.1; 0.4	0.004	0.0047	0.2	−0.1; 0.4	0.218	0.0009

WPPSI-R: Wechsler Primary and Preschool Scale of Intelligence-Revised.

a Regression coefficients, *p*-values, and squared partial correlations (*r* ^2^) for a model including all listed variables, adjusted for child age, sex, and tester.

Partial *r* ^2^ designates the fraction of the variance in IQ explained by the variable when all other variables are held constant.

### Explained Variance

A basic model including only the study design variables (child’s age and tester) and sex explained 7% of the variance in FSIQ (Table 4). Adding parental education and maternal IQ to the model augmented this proportion to 24%. The explained variance of each of the four models of predictor domains ranged between 24% and 27% (Table 4) and was not substantially increased for any of the domains beyond the basic model with study design variables, sex, and core predictors.

**Table 4:** Variance explained (*R* ^2^) by models of predictors.

Statistical model	*R* ^2^		
	FSIQ	VIQ	PIQ
Study variables (child’s age and tester)+sex	0.07	0.03	0.09
Study variables+core predictors (parental education, maternal IQ)+sex	0.24	0.20	0.20
*Parental characteristics*			
Maternal age, paternal age, parity, maternal pre-pregnancy BMI, maternal prenatal marital status*	0.24	0.21	0.20
*Pregnancy and birth*			
Gestational age, birth weight, Apgar score at 5 min., maternal smoking, maternal alcohol consumption*	0.25	0.20	0.21
*Postnatal influences*			
Maternal marital status, breastfeeding, parental smoking, daycare 8+ hrs/day before age 3, separation from parents for 14+ days,irregular breakfast, maternal depression, high parental alcohol intake (>14 drinks/week (women) or >21 drinks/week (men)*	0.26	0.21	0.22
*Postnatal growth*			
Head circumference, height, and BMI at 5 years*	0.27	0.22	0.22
*Final model*			
Maternal age, paternal age, parity, maternal prenatal marital status, birth weight, breastfeeding, irregular breakfast,head circumference and height at 5 years*	0.29	0.25	0.24

* Plus study variables, core predictors, and sex.

The final model accounted for 29% of the variance in FSIQ, indicating a 5% increase over the basic model with study design variables, core predictors and the sex of the child (Table 4). Except for parental education and PIQ, parental education, maternal IQ, and sex of the child were statistically significant predictors of all three IQs in this model, with an adjusted sex difference of 4.9 FSIQ points in favour of girls (*p*<0.001).

Except for birth weight, none of the pregnancy and birth related variables were significantly related to IQ in any model, nor were postnatal marital status, parental smoking, or the remaining indicators of postnatal home environment.

## Discussion

The purpose of this study was to examine the predictive value of a number of variables for IQ at age five years, with particular focus on the contribution of factors beyond maternal IQ and parental education which are both well-known predictors. As expected, maternal IQ, parental education, and sex were significant predictors in all regression models, including the final model that summarized the findings in the four domains of predictors.

This study replicated previous findings of parity [19], birth weight [20], [21], breastfeeding [22], and postnatal growth to be significant predictors of IQ, whereas previously reported predictive patterns were not observed for a number of other variables, including parental age and maternal marital status. In some cases lack of significance most likely reflects correlation with other predictors (e.g., gestational age and birth weight). A thorough evaluation of each association is outside the scope of this paper, but comments on a few of the specific findings are pertinent.

The small, inverse association between maternal BMI and child intelligence found in this study has been reported in a few previous studies [23], [24]. Nutritional factors may play a role, but the association may also be due to residual confounding since the previous studies did not control maternal IQ and associations between obesity and cognitive function is relatively well documented [25]. Thus, the remarkable aspect of the present finding is that the association between maternal BMI and child IQ appears to be independent of both maternal IQ and parental education.

Maternal and paternal age have been associated with offspring cognitive performance, but both negative [26], [27] and positive associations [28] have been reported. In this study the effects of maternal and paternal age were small and restricted to VIQ in the intermediate model.

The finding that verbal IQ scores were higher among children of mothers who were single at the prenatal interview is not consistent with the general finding that single-parent households are negatively associated with cognitive outcomes [19], [29]. This apparent discrepancy may reflect an overrepresentation in our study sample of well-educated and resourceful women [30] who may have chosen single mother status.

Anthropometric measures commonly have been reported as significant correlates of cognitive ability [31]. It has been suggested that postnatal growth predicts IQ better than fetal growth [32] and that the long-term effects of fetal growth may be attenuated substantially by socioeconomic factors and postnatal effects of parental IQ [33]. In this study, birth weight as well as postnatal head circumference and height were significant predictors; the larger partial *r’*s for the postnatal variables, however, indicate that they may be stronger predictors. The quadratic association with head circumference is in line with previous findings of a non-linear association between growth measures and cognitive abilities [20], [34].

The most important finding of this study is the large proportion of variance explained by parental education and maternal IQ. The results corroborate studies showing that these factors are the predominant predictors of the child’s IQ and also suggest that the other factors included in this study may add relatively little explained variance to that of maternal IQ and parental education. Postnatal growth factors were associated with the largest increase in explained variance beyond that explained by maternal IQ and parental education, but these factors may not be predictors in a causal sense but rather correlates of IQ. Thus, maternal IQ and education should be considered mandatory covariates when examining effects of any predictor of intelligence, and lack of adjustment for these factors is likely to bias estimates for other potential predictors and produce a high risk of spurious associations [35].

Longitudinal studies have shown that the balance between different domains of influences may vary noticeably with age at follow-up [3], [36]. While features of the home environment and parent-child interaction are dominant predictors in early childhood, parental education and IQ become increasingly predictive from the age of 2 to 3 years [37]. Differing ages of study samples may therefore contribute to the discrepancies between this and previous studies.

While many previous studies typically included various biomedical or social at-risk groups, the current study sample consisted of essentially healthy children and was likely to include an overrepresentation of well-educated women [30]. This restricted range could potentially result in weaker associations, and it has in fact been suggested that models of predictors fit at-risk samples better than normal samples [38]. However, two studies found a standard set of predictors to predict offspring IQ better among mothers of average IQ than mothers of low IQ [6], [29], and in supplementary stratified and interaction analyses we observed no evidence of substantially different results in subsamples defined by low and high parental education respectively (data not shown). Still, the results of this study should be seen in the context of the study sample which consisted of essentially healthy children of largely well-educated mothers since it is likely that maternal IQ and education will explain more variance in samples characterized by the absence of strong specific, negative influences/risk factors. It should also be born in mind that many developmental factors – such as serious pregnancy and complications, extreme prematurity and substantial maternal alcohol abuse – obviously will affect the individual if they are strong enough. Such cases were, however, rare in the present study sample. This may reflect exclusion criteria or the relatively low participation in the LDPS and the DNBC.

Some further limitations to this study should be noted. First, given the relative importance of proximal factors in early childhood, the lack of a standard measure of proximal home environment, such as parenting style or quality of parent-child interaction, is a genuine limitation. It cannot be ruled out that the inclusion of such a measure would have augmented the fit of the models for this particular age group. Second, measurement of IQ in children as young as five years is subject to situational and non-intellectual factors (e.g. motivation and emotional states) which may dilute the observed associations between predictors and observed IQ. Third, the narrow age-range of the children (60–64 months) may reduce generalizability of the results to older children/adults, considering the limited stability of IQ in early childhood [39].

## Conclusion

This study showed that parental education, maternal IQ, parity, birth weight, breastfeeding, and postnatal growth predicted IQ at 5 years of age, whereas statistically insignificant associations were observed for a number of other variables previously found to be predictive. Parental education and maternal IQ were confirmed as core predictors of IQ, since these predictors were consistently and substantially associated with IQ in all models, whereas the other statistically significant predictors were only associated with a small increase in explained variance. These findings may to some extent reflect the composition of the study sample, but we conclude that the two core predictors must be included as covariates in any study of predictors of intelligence, if residual confounding is to be negligible. This has implications for the design of studies of developmental effects of environmental exposures. In many circumstances, more efforts should be made to obtain high quality measures of maternal IQ and parental education than to obtain information on a large and wide set of covariates, which may be of questionable importance when predicting children’s cognitive development.
